# The effects of simulated video education about falling on falling rate and fear of falling among hospitalized elderly people: a randomized clinical trial

**DOI:** 10.1186/s12912-023-01532-1

**Published:** 2023-10-03

**Authors:** Nasrin Valieiny, Shahzad Pashaeypoor, Sarieh Poortaghi, Farshad Sharifi

**Affiliations:** 1 Department of Community Health and Geriatric Nursing, School of Nursing and Midwifery, Tehran University of Medical Sciences, Tehran, Iran; 2 Elderly Health Research Center, Endocrinology and Metabolism Population Sciences Institute, Tehran University of Medical Sciences, Tehran, Iran

**Keywords:** Falling, Simulation, Video education, Prevention, Elderly people

## Abstract

**Background and aim:**

Management of falling and its consequences is a major challenge of elderly nursing care. An effective educational strategy is essential to prevent falling among elderly people. The aim of this study was to evaluate the effects of simulated video education (SVE) about falling on falling rate and fear of falling (FOF) among hospitalized elderly people.

**Methods:**

This randomized controlled clinical trial was conducted from May 2021 to February 2022. Participants were 132 elderly people conveniently selected from a leading hospital in Qom, Iran, and randomly allocated to an intervention and a control group through block randomization. Data collection instruments were a demographic and clinical questionnaire, a researcher-made falling rate questionnaire, and the Falls Efficacy Scale-International. Participants in the intervention group individually watched three simulated videos (fifteen minutes in total) and had access to the videos for frequent watching. Their FOF was assessed on the first day of hospitalization, hospital discharge, and one and three months after hospital discharge. The data were analyzed at a significance level of less than 0.05 using the SPSS software (v. 16.0).

**Results:**

Groups did not significantly differ from each other respecting baseline demographic and clinical characteristics (P > 0.05). After the intervention, falling rate in the intervention group was 46% less than the control group (incidence rate ratio = 0.5454, 95% CI = 0.307–0.968; P = 0.039). Moreover, the posttest mean score of FOF in the intervention group was significantly less than the control group (P < 0.001).

**Conclusion:**

SVE is effective in significantly reducing falling rate and FOF. Context-based SVE is recommended to reduce falling rate and FOF among hospitalized elderly people.

**Clinical trial registration:**

The effects of simulated video education about falling on falling rate and fear of falling among hospitalized elderly people. Clinical trial registration: this research was registered (17/09/2021) in the https://www.irct.ir with registration number: IRCT20210910052427N1).

## Introduction

Global population is progressively aging due to decreased birth rate and increased life expectancy [1]. The statistics provided by the World Health Organization show that the population of adults above sixty years will reach from 12% to 2015 to 22% in 2050 [2]. The 2016 National Census in Iran also showed that there were more than seven million adults above sixty years in Iran, constituting 9.27% of the total population of the country [3].

Aging is associated with alterations in cognitive status, balance, muscle mass, and muscular strength and coordination and hence, puts elderly people at risk for falling [4–6]. Falling is a sudden and unintentional change in posture to the lower level or the ground [7]. It is a major safety problem in hospitals and its incidence among hospitalized patients in the United States is 700,000 to one million per year. About 30% of all falls are associated with injuries that prolong hospital stays and impose added costs on patients [8].

The final outcomes of falling among elderly people range from complete recovery to death [9]. Falling can lead to bone fractures, head trauma, dependence, depression, anxiety, decreased life expectancy, physical weakness, decreased self-efficacy, limitations in physical activities, reduced quality of life and well-being, increased healthcare costs, and fear of falling (FOF) [10, 11]. FOF is a prevalent problem among elderly people with and without previous fall history and can be associated with immobility. Immobility in turn further weakens the muscles and increases the risk of falling [12, 13].

Almost 78% of all falls in hospital settings are preventable and hence, the implementation of strategies for fall prevention is one of the main goals of elderly care [14]. Elderly patients in hospitals are at risk for falling due to risk factors such as health impairment, comorbid conditions, anesthetic agents, pain, polypharmacy, and muscular weakness, and most of them are unaware of this risk [15].

Patient education is a potentially effective intervention for fall prevention [15]. However, most nurses have limited time for patient education due to their heavy workload. Moreover, the quality of patient education varies among different days of the week and different nurses, depending on their mental states, knowledge and their ability to transfer information. Thus, a cost-effective easy-to-use educational strategy is needed to effectively improve elderly people’s knowledge about falling without increasing nurses’ workload [16].

Simulated video education (SVE) is an innovative method for patient education [17]. It helps frequently repeat learning experiences, transfer standard information, save time and costs, improve learning motivation and learning retention, and facilitate gaining experiences that their direct gain in real world may be risky or difficult [18, 19]. Previous studies reported inconsistent results regarding the effects of video education. For example, a study showed the effectiveness of video education in improving patients’ fall-related knowledge and its insignificant effects on self-efficacy and engagement among hospitalized elders in fall prevention [20]. Another study found that education about fall prevention using audiovisual and written materials had no significant effects on falling rate [21]. Consequently, both these studies highlighted the necessity of further studies in this area. The study was conducted with the aim of evaluating the effects of SVE about falling on falling rate and FOF among hospitalized elderly people, considering the important role of nurses in educating and caring of older individuals, as well as the lack of sufficient studies on cost-effective interventions in fall prevention. The hypothesis of this study posits that SVE is effective in reducing the falling rate and FOF among hospitalized elders.

## Methods

### Design

This randomized controlled clinical trial was conducted from May 2021 to February 2022.

### Participants and setting

The population of this study consisted of elderly people hospitalized in the wards of Shahid Beheshti leading hospital, Qom, Iran. Eligible participants for the study were elderly people above sixty years who had no functional dependence, memory impairment, psychological disorder, and history of participation in fall prevention educational programs, were able to independently walk and move in the ward, and had basic literacy skills ((Information was obtained based on self-report and the patient’s medical record), a hospital stay of more than one week, and a history of at least one fall before hospitalization. Exclusion criteria were patient death, aggravation of health conditions, and transfer to critical care units. Accordingly, 132 hospitalized elderly people were purposefully recruited and randomly allocated to an intervention and a control group through block randomization (with the help of one of the ward nurses). Random allocation sequence was generated using the Randomization.com website and was concealed using 132 opaque envelopes each contained a card labeled with a number based on the generated allocation sequence. One envelope was opened for each participant and the participant was allocated to either groups using the number on the card in the envelope.

Sample size was calculated using the findings of a study [10]. Accordingly, with a type I error of 0.05, a type II error of 0.20, and an FOF posttest mean score of 32.60 ± 8.82 in the control group and 25.50 ± 17.75 in the intervention group, sample size was determined to be 53 per group which was increased to 80 per group considering a potential attrition rate of 30%. The sample size calculation formula was,

$$n=\frac{{\left({Z}_{1-\frac{\alpha }{2}}+{Z}_{1-\beta }\right)}^{2} (2{Pooled \sigma ) }^{2}}{{\left({\mu }_{1}-{\mu }_{2}\right)}^{2}}$$

### Data collection instruments

Instruments were a demographic and clinical questionnaire, a researcher-made falling rate questionnaire, and the Falls Efficacy Scale-International. The items of the demographic and clinical questionnaire were on age, gender, educational level, marital status, employment status, and income, chief complaint, past medical history, medications, length of hospital stay, previous hospitalization history, duration of affliction by the underlying disease, and visual and hearing impairments.

The falling rate questionnaire was a researcher-made questionnaire with items on falling at hospital, frequency of falling at hospital, history of hospitalization due to falling, history of falling at home, and frequency of falling at home.

The Falls Efficacy Scale-International was used for FOF assessment [22, 23].This scale was developed and psychometrically evaluated by Yardley et al., in 2005 in England [23]. It has sixteen items on sixteen activities of daily living. Items are scored on a 1–4 scale as follows: 1: “Not at all concerned”; 2: “Somewhat concerned”; 3: “Fairly concerned”; and 4: “Very concerned”. The possible total score of the scale is 16–64 that higher scores indicate more FOF. Yardley et al., reported that the factor loading values of the items of this scale were 0.659–0.868 and the Cronbach’s alpha and the test-retest correlation coefficient of the scale were 0.96 and 0.96, respectively [23]. Two studies also confirmed the acceptable validity and reliability of the scale and reported that its Cronbach’s alpha and test-retest correlation coefficient were 0.98 and 0.70 [22, 23].

The rate of falls and Falls Efficacy Scale were completed on the day of admission and discharge (in the hospital), one month and three months later (at home).

### Intervention

Study intervention was SVE through three videos made by the first author who worked as a hospital nurse in the study setting. The first video lasted 3:55 min and was on the importance of fall prevention, the outcomes and complications of falls, risk factors of falls, preventability of falls, and fall prevention strategies. The second video, lasted 6:41 min, was on specific fall prevention in the current hospitalization ward and contained materials on wristbands and their colors, prevention of fall from bed through adjusting bed height, use of caster brakes and side rails of the beds, use of nursing call bell, avoidance from sudden out of bed, accurate use of walker and wheelchair, use of appropriate shoes, adequate environmental lighting, use of eye glasses, and other fall-related precautions. The third video, lasted 2:44 min, contained educational materials about the different parts of the ward including nursing station, emergency exit, toilets, wall rails, hand grabs in toilet, nursing call system, and bed lighting system (Table 1). The videos were revised based on the comments of five geriatric nursing specialists and hospital nurses. Participants in the intervention group watched the videos on a cell phone at the presence of the first author and could frequently watch them on their personal cell phones. Several questions were asked from each participant during and after watching the videos. Questions varied according to the intended participant’s health status and literacy level and aimed at ensuring his/her accurate understanding of the educational materials. Participants in the control group received routine care services that included no SVE. All participants in both groups completed all study instruments at the first day of hospitalization and re-completed the Falls Efficacy Scale-International at hospital discharge and one and three months after hospital discharge by telephone and interviewer-administered.

**Table 1:** The contents of simulated video education about prevention of falling

Number of videos	First video	Second video	Third video
Contents	importance of fall prevention, the outcomes and complications of falls, risk factors of falls, preventability of falls, and fall prevention strategies	fall prevention in the current hospitalization ward and contained materials on wristbands and their colors, prevention of fall from bed through adjusting bed height, use of caster brakes and side rails of the beds, use of nursing call bell, avoidance from sudden out of bed, accurate use of walker and wheelchair, use of appropriate shoes, adequate environmental lighting, use of eyeglasses, and other fall-related precautions	educational materials about the different parts of the ward including nursing station, emergency exit, toilets, wall rails, hand grabs in the toilet, nursing call system, and bed lighting system

### Data analysis

The primary and the secondary outcomes of the study were FOF and falling rate, respectively. The data were analyzed using the SPSS software (v. 16.0). The measures of descriptive statistics (namely mean, standard deviation, absolute frequency, and relative frequency) were used to present the data. Statistical methods for data analysis were the Chi-square, independent-sample *t*, and Friedman’s tests (for primary outcome) as well as the analysis of variance, analysis of covariance, and Poisson regression analysis (for secondary outcome). Our approach for analyzing of the data was intention to treat. The Poisson regression analysis was used because the falling rate variable was a counting variable. The level of significance was set at less than 0.05.

## Results

**Fig. 1:**
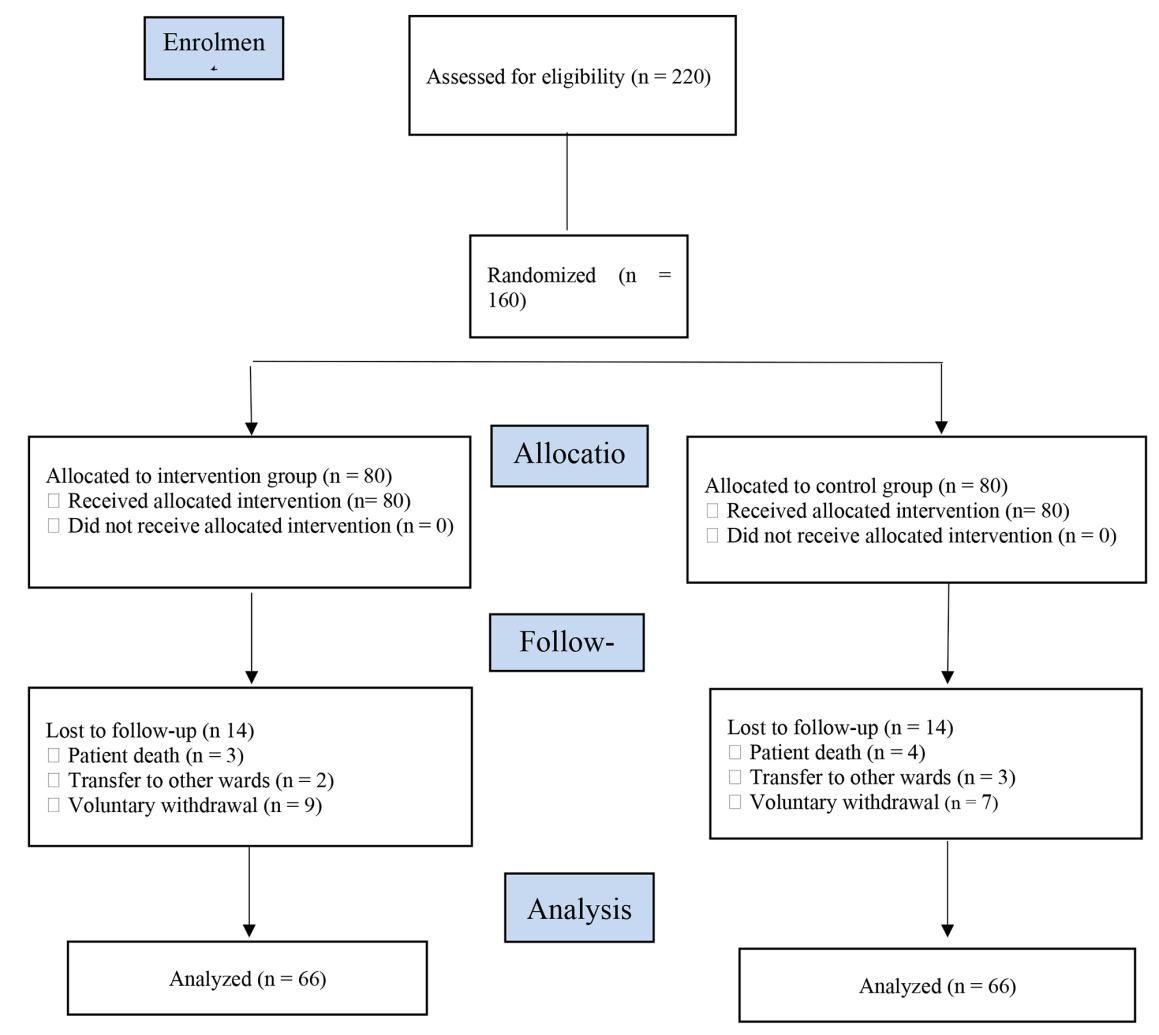
The flow diagram of the study

In total, 132 participants completed the study in two 66-person groups (Fig. 1). The mean of participants’ age was 67.66 ± 6.99 years in the intervention group and 72.48 ± 8.83 years in the control group (P = 0.1). Most participants in these groups were female (69.7% vs. 63.6%) and their income were insufficient (60.6% vs. 62.1%). Moreover, 65.2% of participants in the control group and 45.5% of participants in the intervention group were illiterate. The groups did not significantly differ from each other respecting participants’ demographic and clinical characteristics at baseline (P > 0.05; Table 2).

**Table 2:** Between-group comparisons regarding participants’ characteristics

Characteristics		Control N (%) or Mean ± SD	Intervention N (%) or Mean ± SD	P value
Gender	Male	20 (30.3)	24 (36.4)	0.46*
	Female	46 (69.7)	42 (63.6)	
Educational level	Illiterate	43 (65.2)	30 (45.5)	0.61*
	Primary	17 (25.8)	18 (31.1)	
	High school	5 (7.6)	12 (18.2)	
	Diploma and higher	1 (1.5)	6 (9.1)	
Marital status	Single	0 (0)	2 (3)	0.54*
	Married	38 (57.6)	45 (68.2)	
	Divorced/Widowed	28 (51.4)	19 (28.8)	
Income	Insufficient	40 (60.6)	41 (62.1)	0.80*
	Sufficient	25 (37.9)	23 (34.8)	
	High	1 (1.5)	2 (3)	
Medications	Antihypertensive	48 (72.7)	51 (77.3)	0.54*
	Narcotic	7 (10.6)	7 (10.6)	1.00*
	Analgesic	13 (19.7)	18 (27.3)	0.305*
	Antidepressant	10 (15.2)	13 (19.7)	0.491*
	Antidiabetic	26 (39.4)	28 (42.4)	0.723*
	Anti-inflammatory	34 (51.5)	31 (47)	0.601*
	Antiepileptic	10 (15.2)	9 (13.6)	0.804*
	Antipsychotic	6 (9.1)	4 (6.1)	0.511*
	Others	65 (98.5)	63 (95.5)	0.310*
Hospitalization history	Yes	59 (89.4)	56 (84.8)	0.436*
	No	7 (10.6)	10 (15.2)	
Duration of affliction by the underlying condition	Below three months	42 (63.6)	41 (62.1)	0.875*
	More than three months	24 (36.4)	25 (37.9)	
Visual impairment	Yes	35 (53)	32 (48.5)	0.601*
	No	31 (47)	34 (51.5)	
Hearing impairment	Yes	35 (53)	19 (28.8)	0.51*
	No	31 (47)	47 (71.2)	
Age (Years)		72.48 ± 8.832	67.66 ± 6.99	0.1**
Duration of hospital stay (Days)		9.84 ± 4.85	10.33 ± 4.75	0.56**
Number of medications		4.71 ± 0.67	4.72 ± 0.85	0.910**

*: The results of the Chi-square test; **: The results of the independent-sample *t* test

The results of the research showed that although there was no significant difference in the rates of falls between the intervention and control groups after the intervention, the rate of falls after the intervention decreased clinically (Table 3). But, Poisson regression analysis adjusted for the effects of confounding variables showed that SVE significantly reduced falling rate by 48.26% (P = 0.020). It also revealed that the number of medications had significant effects on falling rate so that each one-point increase in the number of medications significantly increased the risk of falling by 3.133 times (P = 0.045) (Table 4).

**Table 3:** Between- and within-group comparisons respecting the falling rate among hospitalized elderly people

Time Group	Before Number of fall (%)	One month after Number of fall (%)	Three months after Number of fall (%)
Control (N = 66)	12(18.2)	11(16.7)	12(18.2)
Intervention (N = 66)	10(15.2)	6(9.1)	8(12.1)
P value*	0.64	0.194	0.943

*: The results of the Chi-square test

**Table 4:** The results of the Poisson regression analysis for the number of falling rate among hospitalized elderly people

Variables	Incidence rate ratio	95% CI IRR	P value
Group (Intervention/control)	0.4926	0.894–0.271	0.020
Age (each year increased)	0.9843	0.9479–1.0222	0.413
Number of medications (each number increased)	3.133	1.0265–9.5625	0.045
Comorbid conditions	0.9481	0.8267 − 0.0847	0.447
Depression	0.6473	0.3232–1.2998	0.221

The analysis of covariance indicated significant between-group difference respecting the mean score of FOF at hospital discharge and one and three months afterwards (P < 0.001). Friedman’s test also revealed that the variations of the mean score of FOF across the four measurement time points were insignificant in the control group (P = 0.3) and significant in the intervention group (P < 0.001) (Table 5).

**Table 5:** Between- and within-group comparisons respecting the mean score of fear of falling among hospitalized elderly people

Time Group	Before Mean (95% CI)	Hospital discharge Mean (95% CI)	One month after Mean (95% CI)	Three months after Mean (95% CI)
Control	42.273 (42.273–42.273)	42.433 (41.655–43.210)	42.458 (41.294–43.623)	43.979 (42.629–45.230)
Intervention	42.273 (42.273–42.273)	40.916 (40.138–41.694)	39.557 (38.392–40.722)	40.202 (38.852–41.553)
P value*	0.3	0.001	0.001	0.001

*: The results of the Friedman’s test

## Discussion

This study evaluated the effects of SVE about falling on falling rate and FOF among hospitalized elderly people. Findings showed that SVE significantly reduced falling rate. In consistence with this finding, a previous study implemented a comprehensive fall prevention program on gait and balance measurement, medication reconciliation, quality patient education, assessment of adherence to physical exercise, and fall assessment for elderly people every three months for one year [24]. It found that the intervention was effective in significantly improving gait and balance and reducing falling rate and reported that balance improvement through physical exercise, reduction of medication side effects such as hypotension and dizziness through medication reconciliation, knowledge improvement through patient education, and manipulation of environmental risk factors can reduce falling rate [24]. However, a study in Australia found that pamphlet-based and video-based fall prevention education during hospital stay with three-month follow-up telephone contacts after hospital discharge had no significant effects on falling rate [21]. This contradiction may be due to the fact that fall prevention programs are routinely implemented for elderly people in Australia and hence, most elderly people in that country are aware of fall prevention strategies [21].

Our findings also indicated that SVE significantly decreased FOF, particularly one month after the study intervention. However, FOF gradually increased from one month to three months after the intervention. This highlights the necessity of repeating educations to maintain the positive effects of SVE. In agreement with this finding, a study on 72 elderly people showed the significant effects of a collaborative care training program implemented through lecture and group discussion on FOF [25]. Another study assessed the effects of four 30–45-minute education sessions about environmental risk factors of falling, importance of mobility, appropriate use of walking aids, and physical exercises for balance and strength improvement. Educations were provided to one group through role playing and to the other group through videos. That study found that these interventions were effective in significantly reducing FOF by respectively 23.7% and 20.7% and recommended video education as a simple and cost-effective intervention to reduce FOF among elderly people [26]. Contrary to these findings, a study found that education about fall risk factors, fall prevention, individualized standing exercises, and fall assessment and environmental modifications at home had no significant effects on FOF among elderly people [27]. This contradiction may be due to the fact that educations in the present study were provided based on participants’ needs and using simulated videos made based on the actual conditions of the hospitalization wards. Moreover, most elderly people may have limited literacy skills and be unable to use written educational materials, while even illiterate elderly people can independently and frequently use video educations.

Although many studies are needed in this field, according to the discussions, we recommend that the SVE be used more widely in the hospital at the time of admitting the elderly as a simple and cost-effective intervention. But at the same time, it is very important that the educational content is completely adapted to the characteristics of the elderly, including their level of literacy and understanding.

### Limitations

Some participants had problems in understanding the items of the data collection instruments due to their limited literacy skills and hence, we had to complete the instruments for them through the interview method. Moreover, participants were selected from a single hospital and hence, findings should be generalized to other settings with caution. At the same time, the present study has been able to create an effective intervention for the health of the elderly by preparing cost-effective simulated films. Cost-effective interventions are one of the concerns of health planners.

## Conclusion

Preparing educational videos similar to the environment of the inpatient department is a very cost-effective intervention that can lead to greater safety for the elderly in the hospital. Lack of familiarity with the inpatient department, lack of nursing staff in the department, lack of enough time to familiarize the patient with the environment can be covered with the present intervention. This study concludes that SVE about falling is effective in significantly reducing falling rate and FOF. Therefore, healthcare managers and providers can use SVE as an effective method to provide quality education to patients at hospital admission and discharge and thereby, reduce their falling rate and FOF and improve their health. Therefore, it is suggested that further studies should be conducted to investigate the effect of SVE on FOF in community dwelling older adults with larger samples and longer follow-up.

## Data Availability

The datasets used and analyzed during the present study are available from the corresponding author on reasonable request.

## Abbreviations

SVE: Simulated Video Education
FOF: Fear of Falling
